# RMETNet: A cross-subject motor imagery EEG signal classification model based on TSLANet and riemannian geometry features

**DOI:** 10.1371/journal.pone.0347671

**Published:** 2026-04-22

**Authors:** Yun Zhao, Dongyi He, Fudai Ren, Qingling Xia, Linhao Xu, Guanghui Xie, Xiaoling Zhang, Renqiang Yang, Shuaidong Zou, Bin Jiang

**Affiliations:** 1 School of Smart Health, Chongqing Polytechnic University of Electronic Technology, Chongqing, China; 2 School of Artificial Intelligence, Chongqing University of Technology, Chongqing, China; 3 College of Computer Science and Engineering, Chongqing University of Technology, Chongqing, China; Karadeniz Technical University: Karadeniz Teknik Universitesi, TÜRKIYE

## Abstract

Motor imagery electroencephalogram (MI-EEG) analysis is essential for natural interaction and autonomous control in brain-computer interfaces (BCIs). However, deep learning models often struggle with inter-subject variability, which limits their ability to generalize across subjects. This study proposes RMETNet, a novel framework that integrates TSLANet, a spatio-temporal convolution module, and a multi-scale Riemannian geometry feature module. TSLANet suppresses noise and captures complex temporal patterns for preliminary signal decoding, while the spatio-temporal convolution module extracts higher-order representations. The Riemannian branch learns geometry-based distribution features across subjects, and the fused features are used for classification. To address inter-subject distribution shifts, RMETNet incorporates Maximum Mean Discrepancy (MMD) loss for domain adaptation, aligning feature distributions between source and target domains. Experiments show that on the four-class BCI Competition IV 2a (BCICIV2a) dataset, RMETNet achieved accuracies of 71.39% in the cross-subject setting and 80.71% in the subject-dependent setting; on the two-class BCI Competition IV 2b (BCICIV2b) dataset, it achieved 80.93% and 86.76%, respectively. The model consistently outperformed baseline algorithms. Ablation and visualization analyses further validated its effectiveness in reducing inter-subject feature distribution disparities and enhancing MI-EEG decoding. The code is available at: https://github.com/rokanfeermecer486/RMETNet.

## Introduction

Brain-computer interface (BCI) technology enables direct interaction between the human brain and external devices by decoding the electrophysiological signals generated by brain activity, bringing revolutionary breakthroughs to fields such as medical rehabilitation and intelligent control [1]. Among these signals, motor imagery electroencephalography (MI-EEG) is a non-invasive neural signal with high temporal resolution. It is elicited when participants mentally rehearse specific movements, such as left-hand, right-hand, or foot movements, without overt limb motion, thereby activating the motor cortex. This process induces event-related desynchronization (ERD) and event-related synchronization (ERS) in motor-related regions, which are reflected in marked changes in the energy of the μ rhythm (8–12 Hz) and the β rhythm (13–30 Hz) [2]. These characteristics provide an important physiological basis for MI-based BCI research. MI-EEG has become one of the core control signals in BCI systems. Accordingly, accurate MI-EEG decoding is central to efficient brain-computer interaction.

In MI-EEG decoding, the main methods include traditional approaches and deep learning methods. Traditional methods primarily rely on signal processing and shallow machine learning models to analyze EEG signals; however, these methods heavily depend on manually selected features and struggle to capture complex nonlinear relationships [3]. In contrast, deep learning methods, with their robust nonlinear modeling capabilities, have found widespread application in MI-EEG decoding [4]. Among these, convolutional neural networks (CNNs) can effectively extract time-frequency domain features of EEG through their hierarchical structure; recurrent neural networks (RNNs) and their variants, long short-term memory networks (LSTMs), can effectively capture the temporal dynamics of EEG; and models based on the Transformer architecture can significantly enhance the modeling capability of global dependencies in EEG. These models can automatically extract various spatiotemporal features or latent features of MI-EEG, significantly improving EEG decoding accuracy to a certain extent [5]. However, most existing studies focus on subject-dependent models, whereas cross-subject decoding is essential for improving model generalization and supporting broader BCI deployment. Because of substantial neurophysiological heterogeneity across individuals, the same motor imagery task may evoke different EEG response patterns in different subjects. In addition, differences in recording devices and environmental noise further increase the difficulty of cross-subject EEG analysis. As a result, existing models still show limited generalization because they do not effectively capture the relationships between intra-subject and inter-subject features [5]. To address this, researchers have begun to incorporate transfer learning strategies into inter-subject EEG analysis to enhance MI-EEG decoding capabilities. Zhang et al. proposed an adaptive transfer learning algorithm that adjusts pre-trained deep convolutional neural network models to adapt to new target subjects, thereby improving cross-subject decoding performance [6]. Additionally, semi-supervised multi-source transfer learning models have also improved the performance of cross-subject MI-EEG classification tasks to some extent by learning information-rich and domain-invariant representations [7]. Although these methods have made progress in cross-subject EEG decoding, they still show insufficient learning of domain-invariant features and limited robustness to inter-individual EEG noise, which constrains overall decoding performance.

To address these issues, this paper proposes RMETNet (Riemannian MMD-enhanced EEG TSLANet), a neural network designed for cross-subject MI-EEG decoding. First, the Time Series Lightweight Adaptive Network (TSLANet) is introduced to capture long- and short-term interactions in MI-EEG signals while adaptively attenuating high-frequency noise. This module contains an Adaptive Spectral Block (ASB) and an Interactive Convolution Block (ICB), which perform denoising and preliminary temporal decoding. Second, a spatio-temporal convolution module is used to extract higher-level spatio-temporal representations from the TSLANet output. Finally, a multi-scale Riemannian geometry feature module is designed to learn domain-invariant geometric features across subjects, and these features are fused with the convolutional features to improve classification performance. In addition, Maximum Mean Discrepancy (MMD) loss is introduced during training to align source- and target-domain feature distributions, thereby reducing inter-subject variability and improving cross-subject MI-EEG decoding performance.

The main contributions of this study are summarized as follows:

- RMETNet integrates TSLANet, spatio-temporal convolution, and multi-scale Riemannian feature learning to capture complementary temporal, spatial, and geometric information, thereby improving MI-EEG decoding in both subject-dependent and cross-subject settings.
- To better model cross-subject distribution differences, an MMD-loss-based domain adaptation strategy is introduced during training to align source- and target-domain features, reduce inter-subject variability, and improve cross-subject MI-EEG decoding performance.
- The effectiveness of RMETNet was validated on the public BCI Competition IV 2a (BCICIV2a) and BCI Competition IV 2b (BCICIV2b) datasets. On BCICIV2a, the model achieved accuracies of 71.39% in the cross-subject setting and 80.71% in the subject-dependent setting; on BCICIV2b, it achieved 80.93% and 86.76%, respectively. In both evaluation settings, RMETNet outperformed the competing methods.

## Related work

### Subject-dependent MI-EEG decoding

In recent years, deep learning algorithms have demonstrated strong feature extraction capabilities in subject-dependent MI-EEG classification tasks [8]. Researchers have proposed various targeted deep learning architectures based on classical models, such as convolutional neural networks (CNN) [9–11], recurrent neural networks (RNN) [12,13], and hybrid models [14,15], which effectively address the limitations of traditional methods in terms of spatio-temporal feature extraction capabilities. For example, Lawhern et al. [16] proposed a CNN model for analyzing MI-EEG, capable of extracting spectral and temporal features. However, this network is a simple, shallow-layer model with limited feature extraction capabilities. Li et al. [17] utilized CNN to capture dependencies and temporal features between subject-dependent MI-EEG channels and further extracted their higher-order features, effectively improving decoding performance for subject-dependent tasks; Yang et al. [18] constructed a dual-branch CNN model (TBTF-CNN) to simultaneously capture EEG temporal and frequency features, thereby enhancing decoding performance to some extent. Although these models achieved good results in subject-dependent tasks, their adaptability to individual differences is limited, and the features extracted by the dual-branch model may ultimately be integrated through concatenation or simple fusion modules, resulting in limitations in the model’s robustness. Courav et al. [19] utilized the Transformer architecture to model raw EEG signals and explored the impact of input window and convolutional layer parameters on performance, demonstrating the Transformer’s strong generalization capability in small-sample environments. However, the model overly focused on the noise frequency bands of the signal, leading to overfitting in the results. To enhance the modeling capability of complex dynamic features in EEG signals, Ghosh et al. [20] designed a hierarchical network structure by simulating the oscillatory synchronization characteristics of brain neurons, enabling adaptive extraction of dynamic features across different frequency and temporal scales in multi-channel EEG signals. Based on a similar multi-scale modeling approach, Liao et al. [21] integrated a dual-branch structure, improved attention convolutional blocks, and temporal convolutional networks to construct a composite feature extraction framework. Through a multi-level feature fusion strategy, this method not only effectively enhances classification performance in motor imagery tasks but also demonstrates good scalability in terms of computational efficiency. Additionally, Zhang et al. [22] constructed a classification model based on the Inception network, extracting EEG features through a parallel approach combining depth and width, and utilizing residual modules to mitigate the vanishing gradient problem. The model demonstrated potential in cross-subject experiments, but its adaptability to cross-subject tasks was limited due to the absence of a cross-subject training strategy.

Although the aforementioned deep learning methods have improved subject-dependent MI-EEG classification, several limitations remain. Most approaches emphasize temporal or time-frequency characteristics while paying insufficient attention to electrode topology and interactions among brain regions, which restricts model accuracy and robustness in MI-EEG decoding. In addition, some models can capture richer spatio-temporal information but still generalize poorly because they do not fully account for the low signal-to-noise ratio and non-stationarity of MI-EEG signals. As a result, methods designed for subject-dependent settings often fail to align feature distributions effectively across individuals, leading to limited performance in cross-subject decoding.

### Cross-subject MI-EEG decoding

Cross-subject MI-EEG classification tasks are of great significance for improving the generalization ability and robustness of BCI in practical applications. Researchers have begun to introduce transfer learning strategies into inter-subject EEG analysis to alleviate the inconsistency of feature distributions among different subjects and improve MI-EEG decoding ability [23]. For example, Wu et al. [24] proposed a parallel multi-scale filter bank convolutional neural network, which uses hierarchical feature extraction and fine-tuning strategies to construct individualized models on small-sample datasets, thereby significantly improving the adaptability of cross-subject motor imagery classification; EEGSym [25] employs a symmetric network architecture to integrate multi-subject data, effectively mitigating differences in feature distributions across subjects. This method enhances the model’s ability to model common features and improves its generalization performance in cross-subject tasks by introducing a shared parameter mechanism and symmetry loss constraints in the encoder structure. Additionally, a multi-scale adaptive network based on Transformers [26] utilizes a subject adapter module to dynamically fine-tune target data and employs multi-head attention mechanisms to capture signal temporal dependencies, further enhancing the efficiency of cross-subject transfer learning.

Although the aforementioned methods have improved the performance of cross-subject MI-EEG classification to some extent, several key issues remain to be further investigated. First, most algorithms primarily focus on static feature modeling, neglecting the dynamic evolution of EEG signals in the temporal dimension during motor imagery and lacking in-depth modeling of long-term and short-term temporal dependencies. Second, while these methods generally emphasize improving model generalizability, they still fall short in capturing and adapting to individual differences in features. To mitigate the non-stationarity and noise interference of EEG signals, some studies have introduced Riemannian geometry methods [27], which map the covariance matrices of the source domain and target domain to a symmetric positive-definite manifold space, optimizing feature alignment from both statistical and geometric perspectives, and integrating learnable wavelet transforms and Riemannian features into a deep neural network framework. This method demonstrates certain advantages in enhancing cross-subject robustness. However, such models involve high computational complexity during training, limiting their real-time applicability; additionally, over-reliance on a single Riemannian geometric feature makes them susceptible to the non-stationarity and noise fluctuations of EEG signals, leading to unstable covariance estimates. To further enhance cross-subject generalization capabilities, researchers have also introduced strategies based on transfer learning [28]. This type of method effectively enhances the model’s ability in multi-scale feature extraction and cross-domain feature alignment through knowledge transfer between the source domain and the target domain, demonstrating superior cross-subject classification performance on multiple datasets. However, existing transfer learning methods still have shortcomings in spatio-temporal dynamic modeling, cross-domain information fusion, and multi-modal feature collaboration, and further optimization is needed.

To address these challenges, this paper proposes a cross-subject MI-EEG decoding framework that combines TSLANet, spatio-temporal convolution, and domain-adaptive learning. The adaptive spectral and interactive convolution components capture short- and long-term temporal interactions while suppressing high-frequency noise. In addition, the transfer learning framework reduces domain inconsistency between subjects and improves feature alignment across domains. By integrating TSLANet with domain-adaptive learning, the proposed model can jointly handle static distribution shifts and dynamic neural activity patterns during motor imagery, thereby improving MI-EEG decoding performance.

## Methodology

### Overview of RMETNet

**Fig 1:**
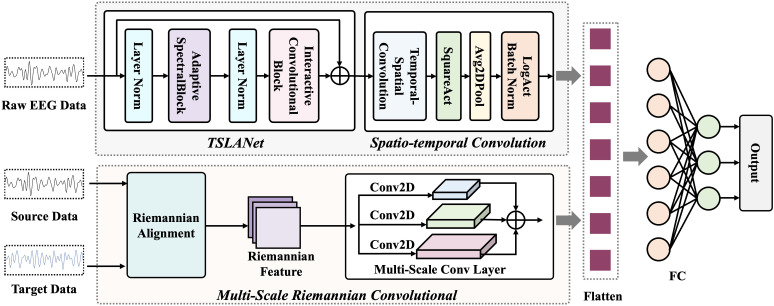
The architecture of RMETNet.

RMETNet is a deep learning framework designed for cross-subject MI-EEG decoding. As shown in Fig 1, it consists of three main modules: (1) TSLANet for temporal feature learning, (2) a spatio-temporal convolution module for higher-level spatio-temporal representation learning, and (3) a multi-scale Riemannian convolution module for cross-subject geometric feature extraction. The inputs include preprocessed raw EEG signals and Riemannian features computed from the source and target domains. The network contains two branches. The first branch takes raw EEG as input, extracts temporal features with TSLANet, and further refines them through temporal and depthwise convolutions. The second branch takes Riemannian features as input and learns geometric representations that characterize cross-subject distribution differences. The features from the two branches are then fused and fed into a fully connected layer with a Softmax classifier for MI-EEG classification. The detailed structure of RMETNet is listed in Table 1.

**Table 1:** RMETNet Structural Details.

Block Name	Type	#Filters/Units	Kernel size	Output	Options
TSLANet	Input	–	–	(1, *C*, *T*)	–
	ASBlock	–	–	(1, *C*, *T*)	Layers = 3
	LayerNorm	–	–	–	–
	ICBlock	–	[2, 4, 8]	(1, *C*, *T*)	Function = GELU, Dropout = 0.5
	Conv1D	–	(1, 4, 1)	–	–
	LayerNorm	–	–	–	–
Spatio-temporal Convolution	Conv2D	*F*_1_	(1, *K*_*E*_)	(*F*_1_, *C*, *T*)	Padding = Same, Maxnorm = 2.0
	Conv2D	$F_{2}=2*F_{1}$	(1, *K*_*E*_)	(*F*_2_, *C*, *T*)	Padding = Same, Maxnorm = 2.0
	Conv2D	$F_{2}=2*F_{1}$	(1, *C*)	(*F*_2_, 1, *T*)	Padding = Valid, Maxnorm = 2.0
	Activation	–	–	(*F*_2_, 1, *T*)	Function = Square
	AvgPool2D	*F*_2_	(1, 75)	(*F*_2_, 1, *T*/75)	Stride = 15
	Activation	–	–	(*F*_2_, 1, *T*/75)	Function = Log
	Dropout	–	–	–	Dropout rate = 0.5
	BatchNorm	–	–	–	–
Multi-scale Riemannian Convolution	Conv2D	*F*_3_	(1, 1)	(*F*_3_, *C*, *C*)	Padding = Same, Maxnorm = 2.0
	Conv2D	*F*_3_	dataset-specific	(*F*_3_, *C*, *C*)	Padding = Same, Maxnorm = 2.0
	Conv2D	*F*_3_	dataset-specific	(*F*_3_, *C*, *C*)	Padding = Valid, Maxnorm = 2.0
	ConCat	–	–	dataset-specific	–
Classifier	LinearLayer	128	–	128	–
	Activation	–	–	–	Function = ReLU
	Dropout	–	–	–	Dropout rate = 0.5
	Softmax	–	–	dataset-specific	–

*Note:* The multi-scale Riemannian convolution module is dataset-dependent. For BCICIV2a, three convolutional layers with kernel sizes 1 × 1, 3 × 3, and 5 × 5 are used. For BCICIV2b, two convolutional layers with kernel sizes 1 × 1 and 2 × 2 are used. Accordingly, the concatenated output dimension and the number of Softmax classes are dataset-dependent. Specifically, for BCICIV2a, the output dimension after concatenation is 3*F*_3_, and the number of Softmax classes is 4; for BCICIV2b, the output dimension after concatenation is 2*F*_3_, and the number of Softmax classes is 2. *C* represents the number of EEG channels, *T* represents the number of time points. In the spatio-temporal convolution module, *K*_*E*_ represents the kernel size of the temporal convolution, which is set to 30 in this paper. *F*_1_, *F*_2_, and *F*_3_ represent the number of filters in the convolutional layers of the spatio-temporal convolution module and the multi-scale Riemannian convolution module, which are set to 12, 24, and 4, respectively. All of these hyperparameters are determined based on empirical judgment and validation on the dataset.

**Fig 2:**
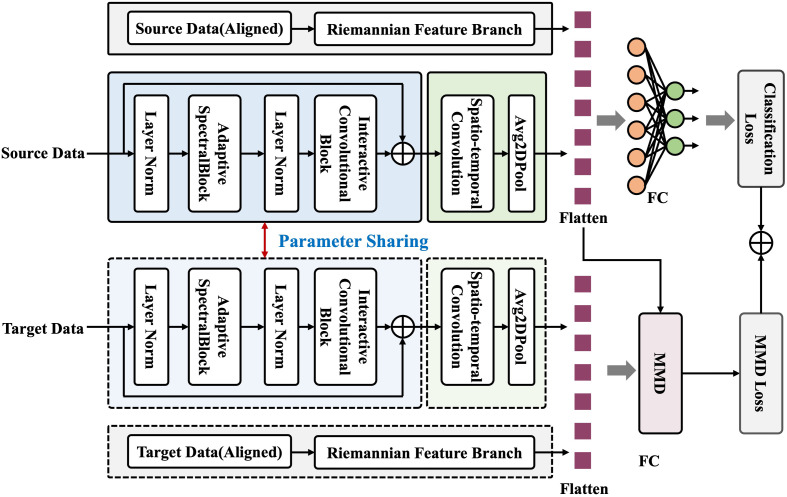
The training process of RMETNet.

More importantly, the proposed model uses Maximum Mean Discrepancy loss (MMD loss) as a domain alignment objective to measure feature distribution discrepancies, as shown in Fig 2. The goal of domain adaptation is to transfer knowledge learned from the source domain to a related target domain by minimizing the discrepancy between their feature distributions. MMD is an unsupervised distribution alignment method that embeds data from the source and target domains into a Reproducing Kernel Hilbert Space (RKHS) and calculates the distance between their mean embeddings in that space to quantify and reduce inter-domain distribution differences. During training, this discrepancy is added to the overall loss as a regularization term. In this way, the global distributions of the two domains are aligned, and cross-domain feature learning is achieved through shared network parameters, as defined below:


$$\min_{f}\left({ℒ}_{cls}+\lambda {ℒ}_{MMD}\right)$$ (1)

where ${ℒ}_{cls}$ represents the classification loss, λ denotes the weighting factor calculated from the source-domain labels, and ${ℒ}_{MMD}$ denotes the MMD loss, which minimizes the distribution discrepancy between the source- and target-domain features. Its calculation formula is as follows:


$${ℒ}_{MMD}={\left\|\frac{1}{m^{2}}\sum_{i=1}^{m}\phi (x_{i})-\frac{1}{n^{2}}\sum_{j=1}^{n}\phi (y_{j})\right\|}_{ℋ}^{2}-2\frac{1}{mn}\sum_{i=1}^{m}\sum_{j=1}^{n}k(x_{i},y_{j})$$ (2)

where *x*_*i*_ and *y*_*j*_ represent the feature representations of the source domain and target domain obtained from the RMETNet, respectively, $\phi (x_{i})$ and $\phi (y_{j})$ are the feature mappings of the source and target domains in the RKHS, *k*(*x*_*i*_, *y*_*j*_) is the kernel function, and *m* and *n* are the number of samples in the source and target domains, respectively. The MMD loss is used to align the feature distributions of the source and target domains, thereby reducing the impact of inter-subject variability on the model.

### TSLANet module

Time-series data are recorded continuously, and each time point contains only limited scalar information; as a result, subtle time-frequency variations in EEG signals are difficult to characterize from single samples alone. Traditional time-frequency analysis methods usually extract only simple temporal descriptors, such as power and phase synchronization, and may therefore miss richer dynamic information [29]. To address this issue, the TSLANet module in RMETNet adopts a hybrid convolutional design that combines spectral modeling with cross-time-frequency interaction, enabling more effective extraction of complex temporal dynamics from EEG signals.

**Fig 3:**
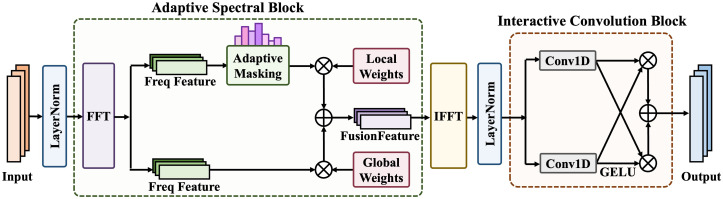
The architecture of TSLANet.

As shown in Fig 3, TSLANet consists of two main components: the Adaptive Spectral Block (ASB) and the Interactive Convolution Block (ICB). The ASB transforms EEG time-series data into the frequency domain, applies adaptive thresholding to suppress high-frequency noise and emphasize informative components, and then reconstructs enhanced time-domain features through the inverse Fourier transform. The ICB is a lightweight convolutional block that uses multiple kernel sizes to refine features interactively, thereby improving the extraction of both local and global temporal patterns. The output of TSLANet is a set of features that jointly encode spectral and temporal information and serve as the input to the subsequent spatio-temporal convolution module.

The TSLANet module processes a multi-channel EEG signal, $X\in {\mathbb{R}}^{N\times M}$, where *N* is the number of channels and *M* is the number of time points. This signal is first passed through an embedding layer to obtain a deep feature representation, $X_{\text{1D}}^{0}\in {\mathbb{R}}^{T\times d_{\text{model}}}$, which serves as the input to the first TSLANet layer. For the *l*-th TSLANet layer, it takes the output from the preceding layer, $X_{\text{1D}}^{l-1}$, and extracts deep temporal variations via a residua*l* connection, formulated as:


$$X_{\text{1D}}^{l}=\text{TSLANet}(X_{\text{1D}}^{l-1})+X_{\text{1D}}^{l-1}$$ (3)

Specifically, the TSLANet module begins by transforming the input features into a comprehensive spectral representation *F* generated by applying a Fast Fourier Transform (FFT) to each channel. To address high-frequency noise in non-stationary EEG signals, the module employs an adaptive filter. This filter first calculates the signal’s Power Spectral Density (PSD), *P* = |*F*|^2^, and then utilizes a learnable threshold, θ, optimized via backpropagation, to dynamically remove noise. This filtering operation is defined by:


$$F_{\text{filtered}}=F⊙(P>\theta )$$ (4)

where (P>θ) is a binary mask, and ⊙ denotes element-wise multiplication. This operation retains the spectral components of *F* that exceed the threshold, effectively filtering out noise while preserving significant features.

To further enrich the features, the module uses a dual-path filter architecture: a global filter *W*_*G*_ and a local filter *W*_*L*_ operate on the original spectrum *F* and the filtered spectrum *F*_filtered_, respectively, then these two filters are fused through element-wise addition:


$$F_{\text{integrated}}=W_{G}⊙F+W_{L}⊙F_{\text{filtered}}$$ (5)

where *W*_*G*_ and *W*_*L*_ are learnable parameters that adaptively adjust the contributions of the global and local filters.

The integrated features are subsequently reconstructed back into the time domain via an Inverse Fast Fourier Transform (IFFT), resulting in an enhanced signal:


$$X_{\text{1D}}^{\prime }=\text{IFFT}(F_{\text{integrated}})\in {\mathbb{R}}^{C\times L^{\prime }}$$ (6)

Finally, this enhanced time-domain representation, $X_{\text{1D}}^{\prime }$, is fed into an interactive convolutional module designed to capture both local details and long-range temporal dependencies. This module uses a convolutional layer with a small kernel (Conv1D_1_) in parallel with a layer having a large kernel (Conv1D_2_), interacting through a gating mechanism:


$$A_{1}=\phi ({\text{Conv1D}}_{1}(X_{\text{1D}}^{\prime }))⊙{\text{Conv1D}}_{2}(X_{\text{1D}}^{\prime })$$ (7)

$$A_{2}=\phi ({\text{Conv1D}}_{2}(X_{\text{1D}}^{\prime }))⊙{\text{Conv1D}}_{1}(X_{\text{1D}}^{\prime })$$ (8)

where ϕ is the GELU activation function. The summed output of these two branches is passed through a final convolutional layer to produce the module’s final output, *X*_out_, which represents the $\text{TSLANet}(X_{\text{1D}}^{l-1})$ term in the residual connection:


$$X_{\text{out}}={\text{Conv1D}}_{3}(A_{1}+A_{2})$$ (9)

### Spatio-temporal convolution module

To enable the model to learn local temporal features, two sequential convolutional layers are employed. A two-dimensional convolutional kernel of size (1, *K*_*e*_), denoted as $S_{K_{e}}^{1}$, is used to capture temporal information. Additionally, a two-dimensional convolutional kernel of size (*C*, 1), denoted as $S_{1}^{C}$, is applied to capture spatial information, where *C* corresponds to the number of EEG channels. The spatial convolution serves to learn spatial filters (i.e., correlations among EEG channels), effectively extracting local features in the temporal dimension and global features in the spatial dimension from the EEG signals.

Let *X*_1D_ denote the 1D feature sample output by the TSLANet module. The output is reshaped into a 2D sample as follows:


$$X_{\text{2D}}=\text{ExpandDim}(X_{\text{1D}},1)$$ (10)

where ExpandDim(*,1) denotes the expansion operation along the second dimension, i.e., transforming the input from $X\in {\mathbb{R}}^{b\times c\times s}$ to $X\in {\mathbb{R}}^{b\times 1\times c\times s}$ so that the EEG features can match the 2D convolutional kernels.

To preserve the non-stationary characteristics of EEG signals, a square activation function is applied. To reduce noise and the number of model parameters, a pooling layer is used to downsample the feature maps. Furthermore, to normalize the mean of the latent features to zero, thereby improving training stability and accelerating the convergence process [30], batch normalization is applied after the activation function. The output of the convolution operation, denoted as $X_{\text{2D}}\in {\mathbb{R}}^{b\times f\times c\times s}$, where *b* is the number of samples, *f* is the number of output feature maps, *c* is the number of channels, and *s* is the number of feature points per sample. The operations are formally defined as:


$${\dot{X}}_{\text{2D}}=\text{Conv2D}(\text{Conv2D}(X_{\text{2D}},S_{K_{e}}^{1}),S_{K_{e}}^{1})$$ (11)

$$X_{\text{2D}}^{\prime }=\text{AvgPool}(\text{Square}(\text{Conv2D}({\dot{X}}_{\text{2D}},S_{1}^{C})))$$ (12)

where $S_{K_{e}}^{1}$ and $S_{1}^{C}$ represent the kernel sizes of the two convolution operations. Conv2D() denotes the two-dimensional convolution operation, Square() denotes the square activation function, and AvgPool() denotes average pooling.

To ensure the stability of feature distributions and to avoid internal covariate shift within the model, a logarithmic activation function and batch normalization are applied after the square activation, as defined below:


$$X_{\text{2D}}=\text{BatchNorm}(\log(X_{\text{2D}}^{\prime }))$$ (13)

where $X_{\text{2D}}^{\prime }$ is the output feature map after the three convolution and pooling operations, log() denotes the logarithmic activation function, and BatchNorm() denotes batch normalization.

### Multi-scale riemannian convolution module

Riemannian geometry provides an effective framework for aligning covariance-based EEG representations on a symmetric positive-definite manifold. After defining an appropriate metric, the mapped features preserve both global structure and inter-channel relationships, which helps reduce distribution differences across subjects and domains. For EEG signal processing, this study computes covariance matrices from the source and target domains and maps them into Riemannian space to obtain aligned feature representations. This process captures both global and local structural information in EEG signals and improves the model’s generalization ability in cross-subject learning.

The input to the Riemannian convolution module is the raw EEG data, which is first processed to obtain the covariance matrix of the source domain and target domain data. The covariance matrix is calculated as follows:


$$Cov=\frac{1}{N}\sum_{i=1}^{N}(X_{i}-\mu )(X_{i}-\mu {)}^{T}\in {\mathbb{R}}^{C\times C}$$ (14)

where *X*_*i*_ is the *i*-th sample of the EEG signal, μ is the mean of the samples, and *N* is the number of samples. The covariance matrix captures the second-order statistics of the EEG signal, reflecting the relationships between different channels.

Next, the Riemannian distance between the covariance matrices of the source and target domains is computed as follows:


$$d_{R}(Cov_{1},Cov_{2})=\|log(Cov_{1})-\log(Cov_{2}){\|}_{F}$$ (15)

where *Cov*_1_ and *Cov*_2_ are the covariance matrices of the source and target domains, respectively, $\|\cdot {\|}_{F}$ denotes the Frobenius norm, and the matrix logarithm maps the covariance matrices into Riemannian space, where the distance is measured.

To align all covariance matrices to a reference point in Riemannian space—commonly the geometric mean $\bar{\text{Cov}}$ of all matrices—we project each covariance matrix ${\text{Cov}}_{i}$ to the tangent space at $\bar{\text{Cov}}$ via:


$$\tilde{Cov}={\overset{―}{Cov}}^{-\frac{1}{2}}Cov_{i}{\overset{―}{Cov}}^{-\frac{1}{2}}$$ (16)

To enhance the robustness of the aligned covariance matrices and extract multi-scale features, three convolutional layers with different kernel sizes are used to capture local, mid-range, and global structures. These extracted features are subsequently fused, flattened, and projected into a compact feature space:


$$X_{cat}=Concat\left(Conv_{1}(\tilde{Cov}),Conv_{2}(\tilde{Cov}),Conv_{3}(\tilde{Cov})\right)$$ (17)

$$X_{rout}=Linear\left(Flatten(X_{cat})\right)$$ (18)

The output of this multi-scale Riemannian module is then fused with the features extracted from the time-frequency convolution module and passed to the classifier.

The dimensionality of the Riemannian geometric features depends on the number of EEG channels, so the module configuration differs slightly across datasets. For the BCICIV2a dataset, the three convolutional kernel sizes are set to 1 × 1, 3 × 3, and 5 × 5, respectively. For the BCICIV2b dataset, two convolutional layers with kernel sizes of 1 × 1 and 2 × 2 are used.

### Classifier module

The classifier module consists of a fully connected layer and a Softmax layer. The fully connected layer takes the concatenated features from the TSLANet module and the multi-scale Riemannian convolution module as input, applies a linear transformation, and outputs a feature vector of size 128. This is followed by a ReLU activation function to introduce non-linearity. A dropout layer with a dropout rate of 0.5 is applied to prevent overfitting. Finally, the Softmax layer outputs the classification probabilities for each class. Then, the Cross-Entropy loss function is used to calculate the classification loss, which is defined as:


$${ℒ}_{cls}=-\frac{1}{N}\sum_{i=1}^{N}\sum_{j=1}^{C}y_{ij}\log({\hat{y}}_{ij})$$ (19)

where *N* is the number of samples, *C* is the number of classes, *y*_*ij*_ is the true label for sample *i* and class *j*, and ${\hat{y}}_{ij}$ is the predicted probability for sample *i* and class *j*. The model is trained to minimize this loss function.

## Experimental setup

Two publicly available motor imagery EEG datasets, summarized in Table 2, were used to evaluate the proposed model under both subject-dependent and cross-subject settings. We compared RMETNet with several state-of-the-art methods, conducted ablation studies, and used visualization analyses to examine the learned feature distributions.

**Table 2:** The summary of the BCICIV2a and BCICIV2b datasets used in this paper.

Datasets	Subjects	Channels	Classes	Trials	Samples	Sampling Rate
BCICIV2a [31]	9	22	4	288 × 9	1000	250
BCICIV2b [32]	9	3	2	480 × 9^1^	1000	250

^1^For BCICIV2b, only the last three feedback sessions were used for all experiments, resulting in 480 trials per subject.

### Datasets

The BCICIV2a [31] and BCICIV2b [32], provided by Graz University of Technology, are widely used benchmarks for motor imagery-based EEG classification tasks. BCICIV2a contains EEG recordings from nine subjects performing four motor imagery tasks: left hand, right hand, both feet, and tongue. The EEG signals were recorded using 22 Ag/AgCl electrodes at a sampling rate of 250 Hz. Data were collected in two sessions at different times, with each session containing 288 trials (72 trials per class). BCICIV2b also includes recordings from nine subjects but focuses on two motor imagery tasks: left hand and right hand. EEG data were collected using 3 channels at the same sampling rate of 250 Hz. This dataset consists of five sessions: the first two sessions, without feedback, contain 120 trials in total (60 trials per class), while the last three sessions, with feedback, comprise 480 trials (160 per session, 80 per class). To remain consistent with prior studies, in this study, only the last three feedback sessions were used for all experiments on BCICIV2b. Therefore, each subject contributed 480 trials in our experiments. In addition, the original BCICIV2a and BCICIV2b recordings had already undergone acquisition-stage preprocessing, including a 0.5–100 Hz band-pass filter and a 50 Hz notch filter [31,32].

**Fig 4:**
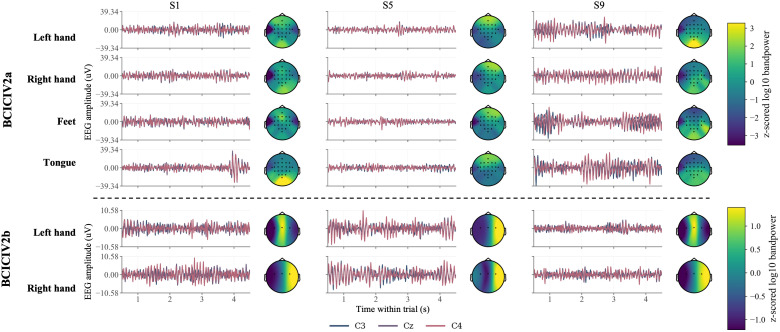
Visualization of representative motor-imagery trials from the BCICIV2a and BCICIV2b datasets. The top panel shows BCICIV2a and the bottom panel shows BCICIV2b; columns correspond to subjects 1, 5, and 9. For BCICIV2a, rows represent left-hand, right-hand, feet, and tongue imagery, respectively; for BCICIV2b, rows represent left-hand and right-hand imagery. In each subpanel, the left plot shows EEG waveforms from channels C3, Cz, and C4 over the trial interval from 0.5 to 4.5 s, whereas the right plot shows the scalp topography computed from the same interval using band power in the 8-30 Hz range. Topographic values are expressed as z-scored log10 band power.

To visualize the characteristics of the two datasets, representative motor imagery trials from both BCICIV2a and BCICIV2b are shown in Fig 4. For each dataset, three subjects (subjects 1, 5, and 9) were selected to illustrate cross-subject variability, and for each subject, one trial from each motor imagery class was randomly chosen. The EEG waveforms from channels C3, Cz, and C4 were plotted over the interval from 0.5 to 4.5 s after cue onset, which is the analysis window used in this study. In addition, scalp topographies were computed from the same interval using band power in the 8–30 Hz range, with values expressed as z-scored log_10_ band power to highlight the spatial patterns of task-related neural activity. As can be observed, the temporal waveforms and spatial distributions vary considerably across subjects, indicating substantial inter-subject variability that poses a major challenge for cross-subject classification. Meanwhile, EEG patterns elicited by different motor imagery tasks often differ only subtly in both temporal and spatial characteristics, which increases inter-class similarity and makes discriminative feature extraction and robust classification more difficult.

### Data preprocessing and division

The original EEG samples have dimensions of *ch* × *sp*, where *ch* denotes the number of electrode channels and *sp* denotes the number of temporal samples. Specifically, *ch* = 22 for BCICIV2a and *ch* = 3 for BCICIV2b. For each trial, a fixed EEG segment from 0.5 s to 4.5 s after cue onset was extracted for analysis. Thus, the segment length was 4 s, corresponding to 1000 temporal samples at a sampling rate of 250 Hz. The same time window was used for both BCICIV2a and BCICIV2b. No overlapping windows or trial-level augmentation were used. The extracted EEG signals were directly used for subsequent processing, and Z-score normalization was applied to reduce signal variability and improve training stability:


$$x_{o}=\frac{x_{i}-\mu }{\sigma }$$ (20)

where *x*_*i*_ and *x*_*o*_ represent the raw and normalized data, respectively. The mean μ and standard deviation σ are computed from the training set and directly applied to the test set to maintain consistency during model training and evaluation.

Covariance matrices were computed separately for the training and validation sets, and Riemannian alignment was applied to reduce inter-subject variability and improve model generalization. Accordingly, the inputs for the cross-subject experiments included both raw EEG signals and Riemannian-aligned features. For cross-subject evaluation, a leave-one-subject-out (LOSO) strategy was adopted. In each fold, all available trials from one subject were used as the test set, while the data from the remaining subjects were used for training and validation. For BCICIV2b, only the three feedback sessions were included, resulting in 480 trials per subject. For subject-dependent evaluation, each subject was processed independently. Only the data from the same subject were used, and they were further divided into training, validation, and test sets with a ratio of 8:1:1.

### Evaluation metrics

To evaluate the performance of the proposed model, this paper uses two commonly used metrics in the field of motor imagery EEG classification, including accuracy (ACC) and kappa coefficient (KAPPA). The accuracy is defined as the ratio of correctly classified samples to the total number of samples, while the kappa coefficient is a measure of inter-rater agreement that accounts for chance agreement. These metrics are calculated as follows:


$$ACC=\frac{TP+TN}{TP+TN+FP+FN}$$ (21)

where *TP*, *TN*, *FP*, and *FN* represent the true positives, true negatives, false positives, and false negatives, respectively.


$$KAPPA=\frac{P_{o}-P_{e}}{1-P_{e}}$$ (22)

where *P*_*o*_ is the observed agreement and *P*_*e*_ is the expected agreement by chance. The kappa coefficient ranges from −1–1, where 1 indicates perfect agreement, 0 indicates no agreement beyond chance, and negative values indicate less than chance agreement.

### Implementation details

The proposed model was implemented in PyTorch with Python 3.8 and trained on an NVIDIA GeForce RTX 4080 SUPER The batch size is set to 64, with a total of 500 training epochs. The Adam optimizer is used with a learning rate of 0.0001 and a weight decay of 0.075. The TSLANet module consists of three layers. To ensure proper convergence during training, L2 regularization is applied to prevent overfitting. During the training process, the model weights corresponding to the lowest validation loss are saved, and these weights are loaded for testing.

## Results and discussion

### Comparison with state-of-the-art methods

To evaluate the feature recognition performance of RMETNet on MI-EEG signals, we conducted both subject-dependent and cross-subject experiments. Several representative state-of-the-art methods were selected as baselines for systematic comparison on the two public datasets. The baseline methods are briefly described below:

- **FBCSP [33]:** A traditional machine learning method, its principle is based on implementing a spatial feature extraction algorithm using Common Spatial Pattern (CSP) on partitioned frequency bands, combined with a feature selection algorithm. Specifically, the frequency band is first sliced, then CSP filtering is applied to each sub-band. Finally, features are selected from the filtered signals to produce the classification result.
- **EEGNet [16]:** A compact convolutional neural network that learns temporal filters via convolutional kernels to capture motion-related frequency information. The network also employs a separable convolution structure, including Depthwise and Pointwise Convolutions, to learn spatial filters. This design significantly reduces the number of model training parameters while preserving robust feature extraction capabilities.
- **ShallowConvNet [3]:** The data transformation process of ShallowConvNet bears similarity to the feature extraction method of FBCSP, with its core lying in the joint modeling of temporal and spatial features. Specifically, ShallowConvNet extracts spatio-temporal features through a temporal convolution and spatial filters, followed by the application of a squaring nonlinearity, a mean pooling layer, and a logarithmic activation function to further extract deep features and enhance their discriminability.
- **EEG-TCNet [34]:** Proposed as an extension of EEGNet, EEG-TCNet introduces a Temporal Convolutional Network (TCN) structure after EEGNet’s initial feature extraction to further mine information in the temporal dimension. This design combines the efficient feature extraction of EEGNet with TCN’s capacity for modeling long-term dependencies, thereby enhancing the representational power for temporal characteristics of EEG signals and improving classification performance.
- **EEG-ITNet [35]:** This model introduces Inception modules and dilated causal convolutions to efficiently extract rich spectral, spatial, and temporal features from multi-channel EEG signals. The Inception modules capture features from different frequency bands through multi-scale convolutions, while the dilated causal convolutions model long-term dependencies by leveraging an expanded receptive field, thus comprehensively enhancing the feature representation and classification performance for EEG signals.
- **DS-TKL [36]:** This method achieves cross-domain learning by selecting discriminative features from the source domain and performing pseudo-label correction in the target domain. The DS-TKL method first preprocesses samples through centroid alignment to reduce the distribution discrepancy between the source and target domains. Subsequently, it employs Riemannian tangent space features for feature adaptation to further promote feature space alignment between the domains. During the feature adaptation process, a dual selection is implemented through a regularization mechanism to optimize the feature selection process, significantly improving classification performance during iteration.
- **Transformer-Based Method [37]:** This method proposes an end-to-end EEG signal decoding algorithm based on swarm intelligence theory and virtual adversarial training, which integrates the Transformer mechanism to optimize traditional convolutional classification methods. By introducing a self-attention mechanism, the research aims to expand the receptive field for EEG signals to capture global dependencies, thereby capturing broader spatio-temporal features. This approach trains the neural network by optimizing the model’s global parameters, enhancing the model’s global feature learning ability while improving EEG signal classification performance.
- **EEGConformer [38]:** This is a compact, EEG-based Transformer architecture. Its principle involves using convolutional modules to learn local one-dimensional temporal and spatial features. The advantage of this model lies in its subsequent use of a self-attention module to extract global dependencies within these local temporal features. Compared to previous global temporal feature extraction strategies, the features learned by a model incorporating a self-attention mechanism are more comprehensive, achieving higher model performance.

In this paper, accuracy and the Kappa coefficient are used as metrics to quantitatively evaluate the proposed model and compare it with the baseline classification methods.

### Results on BCICIV2a dataset

Table 3 summarizes the classification accuracy and Kappa scores of the proposed RMETNet and several representative deep learning models on the BCICIV2a dataset under the subject-dependent setting. To ensure a fair comparison of feature extraction capability, the cross-domain modules, including MMD loss and Riemannian geometry feature fusion, were removed, and only the base network was evaluated. As shown in the table, RMETNet achieves the best overall performance, reaching an average accuracy of 80.77% and a Kappa value of 0.76. In addition, it obtains the highest accuracies on four subjects (S01, S03, S04, and S07), while maintaining competitive performance on the remaining subjects, indicating strong subject-dependent decoding capability.

**Table 3:** Subject-dependent classification results on the BCICIV2a dataset. The best results are highlighted in bold.

Methods	S01	S02	S03	S04	S05	S06	S07	S08	S09	Avg.	Kappa
EEGNet [16]	84.34	54.06	87.54	63.59	67.39	54.88	88.80	76.75	74.24	72.40±12.51^**^	0.63
ShallowConvNet [3]	79.51	56.25	88.89	80.90	57.29	53.82	91.67	81.25	79.17	74.31±13.71^**^	0.66
EEG-TCNet [34]	85.77	65.02	94.51	64.91	**75.00**	61.40	87.36	83.76	78.03	77.31±10.92	0.71
EEG-ITNet [35]	84.38	62.85	89.93	69.10	74.31	57.64	88.54	83.68	80.21	76.74±10.82^*^	0.71
EEGConformer [38]	88.19	61.46	93.40	78.13	52.08	**65.28**	92.36	**88.19**	**88.89**	78.66±14.43	0.71
LMDANet [39]	86.50	**67.40**	91.70	77.40	65.60	61.10	91.30	83.30	85.40	78.86±10.88	0.71
RMETNet (ours)	**88.31**	59.88	**95.56**	**80.20**	71.59	62.31	**96.33**	85.38	87.40	**80.77±12.67**	**0.76**

The symbols ^*^ and ^**^ indicate statistically significant differences compared to the proposed model at the *p* < 0.05 and *p* < 0.01 levels, respectively, based on paired Wilcoxon signed-rank tests.

Compared with classical CNN-based approaches, RMETNet improves the average accuracy by 8.37 and 6.46% over EEGNet and ShallowConvNet, respectively. More importantly, the paired Wilcoxon signed-rank test shows that these improvements are statistically significant at the *p* < 0.01 level, demonstrating the clear advantage of the proposed model over conventional lightweight convolutional architectures in four-class motor imagery classification. RMETNet also outperforms EEG-ITNet by 4.03%, and this improvement is statistically significant at the *p* < 0.05 level, further confirming the effectiveness of the proposed architecture. In comparison with EEG-TCNet, EEGConformer, and LMDANet, RMETNet still achieves the highest average accuracy, with gains of 3.46, 2.11, and 1.91%, respectively, although no statistically significant difference is observed according to the reported test results. Overall, these findings indicate that RMETNet can learn more discriminative spatio-temporal representations and provides superior performance under the subject-dependent evaluation protocol.

As shown in Table 4, RMETNet achieves the best performance in the cross-subject setting, obtaining an average classification accuracy of 71.39% and a Kappa value of 0.59. Notably, these results are achieved using the same network configuration and hyperparameter settings for all target subjects, which further demonstrates the robustness of the proposed framework. RMETNet attains the highest accuracies on seven out of nine subjects (S01, S03, S04, S05, S07, S08, and S09), showing strong resistance to inter-subject variability.

**Table 4:** Cross-subject classification results on the BCICIV2a dataset. The best results are highlighted in bold.

Methods	S01	S02	S03	S04	S05	S06	S07	S08	S09	Avg.	Kappa
EEGNet [16]	65.53	54.06	73.42	53.10	61.04	49.69	66.87	63.12	74.24	62.34±8.24^*^	0.27
ShallowConvNet [3]	70.51	46.25	71.22	50.65	62.44	49.07	64.72	67.30	62.53	60.52±8.93^**^	0.35
EEG-TCNet [34]	67.40	**62.94**	79.90	49.44	60.00	51.60	71.52	71.11	69.12	64.78±9.28	0.45
EEG-ITNet [35]	68.13	56.30	75.87	55.21	65.40	56.89	74.30	67.66	67.00	65.20±7.17^*^	0.46
Transformer-Based [37]	61.61	59.12	64.35	62.36	67.35	**63.02**	65.62	63.67	64.97	63.56±2.27	0.46
RMETNet (ours)	**76.01**	49.79	**84.58**	**64.93**	**67.78**	61.18	**80.00**	**80.73**	**77.54**	**71.39±10.65**	**0.59**

The symbols ^*^ and ^**^ indicate statistically significant differences compared to the proposed model at the *p* < 0.05 and *p* < 0.01 levels, respectively, based on paired Wilcoxon signed-rank tests.

In terms of average accuracy, RMETNet surpasses EEGNet, ShallowConvNet, EEG-TCNet, EEG-ITNet, and the Transformer-based model by 9.05, 10.87, 6.61, 6.19, and 7.83%, respectively. According to the paired Wilcoxon signed-rank test, the improvements over EEGNet and EEG-ITNet are statistically significant at the *p* < 0.05 level, while the improvement over ShallowConvNet is statistically significant at the *p* < 0.01 level. These results indicate that classical and conventional deep learning baselines still face substantial difficulty in handling cross-subject EEG distribution shifts. Although RMETNet also achieves higher average accuracies than EEG-TCNet and the Transformer-based model, the corresponding differences are not marked as statistically significant. Taken together, the above results suggest that RMETNet is more effective at learning transferable representations, reducing cross-subject distribution discrepancy, and improving the generalization performance of motor imagery EEG decoding.

### Results on BCICIV2b Dataset

The BCICIV2b dataset is a widely used benchmark for three-channel, binary motor imagery classification and therefore provides an appropriate testbed for evaluating the effectiveness of RMETNet in a low-channel scenario. As reported in Table 5, RMETNet achieves the best overall performance in the subject-dependent setting, with an average classification accuracy of 86.76% and a Kappa value of 0.74. Moreover, RMETNet obtains the best results on five of the nine subjects (S2, S3, S4, S5, and S7) and reaches a peak accuracy of 98.75% on subject S4.

**Table 5:** Subject-dependent classification results on the BCICIV2b dataset. The best results are highlighted in bold.

Methods	S1	S2	S3	S4	S5	S6	S7	S8	S9	Avg	Kappa
FBCSP [33]	70.00	60.36	60.94	97.50	93.12	80.63	78.13	92.50	86.88	80.01±13.06^**^	0.60
EEGNet [16]	75.94	57.64	58.43	98.13	81.25	88.75	84.06	93.44	89.69	80.81±13.64	0.61
ShallowConvNet [3]	74.30	56.00	57.50	97.50	95.30	82.10	79.60	87.50	86.50	79.59±13.97^**^	0.59
EEGConformer [38]	**82.50**	65.70	63.70	98.40	86.50	**90.30**	87.80	94.30	91.10	84.48±11.41	0.69
LMDANet [39]	81.20	62.10	71.80	98.40	95.60	89.30	85.00	**95.60**	**91.80**	85.64±11.44	0.71
RMETNet (ours)	76.88	**67.08**	**81.88**	**98.75**	**95.94**	85.31	**92.81**	93.44	88.75	**86.76±9.57**	**0.74**

The symbols ^*^ and ^**^ indicate statistically significant differences compared to the proposed model at the *p* < 0.05 and *p* < 0.01 levels, respectively, based on paired Wilcoxon signed-rank tests.

Compared with FBCSP, EEGNet, ShallowConvNet, EEGConformer, and LMDANet, RMETNet improves the average accuracy by 6.75, 5.95, 7.17, 2.28, and 1.12%, respectively. Among these comparisons, the gains over FBCSP and ShallowConvNet are statistically significant at the *p* < 0.01 level according to the paired Wilcoxon signed-rank test, highlighting the clear superiority of RMETNet over traditional feature-engineering methods and shallow convolutional baselines. Although RMETNet also achieves higher average accuracy than EEGNet, EEGConformer, and LMDANet, these differences are not marked as statistically significant in the current results. In addition, RMETNet exhibits the smallest standard deviation (9.57), indicating more stable performance across subjects. These findings demonstrate that the proposed model remains highly effective even with only three EEG channels and can extract discriminative task-related features for binary motor imagery decoding more reliably than competing methods.

Furthermore, the robustness and generalization ability of the proposed model were evaluated through cross-subject experiments on the BCICIV2b dataset, with the results shown in Table 6. In this more challenging setting, RMETNet again achieves the best overall performance, yielding an average classification accuracy of 80.93% and a Kappa value of 0.54. More importantly, RMETNet ranks first on eight of the nine subjects, and only on subject S4 does it perform slightly below the best competing method, while still achieving a high accuracy of 91.63%. This demonstrates that RMETNet can maintain strong and consistent decoding performance under substantial inter-subject distribution differences.

**Table 6:** Cross-subject classification results on the BCICIV2b dataset. The best results are highlighted in bold.

Methods	S1	S2	S3	S4	S5	S6	S7	S8	S9	Avg	Kappa
EEGNet [16]	55.06	49.34	56.02	75.88	61.70	58.12	59.95	60.26	60.05	59.60±6.76^**^	0.34
ShallowConvNet [3]	61.84	51.60	**62.75**	73.22	62.15	56.89	62.81	68.92	71.26	63.49±6.45^**^	0.39
DS-TKL [36]	73.00	59.75	60.00	**92.75**	71.00	71.50	67.25	67.25	72.25	70.53±9.14^**^	∼
RMETNet (ours)	**81.88**	**73.21**	66.70	91.63	**85.74**	**83.36**	**86.90**	**81.71**	**77.20**	**80.93±7.12**	**0.54**

The symbols ^*^ and ^**^ indicate statistically significant differences compared to the proposed model at the *p* < 0.05 and *p* < 0.01 levels, respectively, based on paired Wilcoxon signed-rank tests.

From the perspective of average accuracy, RMETNet improves upon EEGNet, ShallowConvNet, and DS-TKL by 21.33, 17.44, and 10.40%, respectively. The paired Wilcoxon signed-rank test further shows that all these improvements are statistically significant at the *p* < 0.01 level, providing strong evidence for the superiority of RMETNet in cross-subject decoding on the BCICIV2b dataset. In addition, RMETNet achieves a lower standard deviation (7.12) than DS-TKL (9.14), indicating better stability across subjects. These results suggest that RMETNet is capable of learning more transferable and domain-robust EEG representations, thereby effectively alleviating the negative impact of inter-subject variability. Such a property is particularly important for practical BCI systems, where reducing calibration effort and improving generalization to unseen users are key requirements.

Overall, the statistical analysis based on paired Wilcoxon signed-rank tests demonstrates that the proposed RMETNet not only achieves the highest average accuracies on both datasets, but also exhibits statistically significant improvements over several representative baselines, especially in the more challenging cross-subject setting. These results suggest that the proposed model can effectively learn more discriminative and transferable representations for motor imagery EEG decoding, thereby improving the generalization performance across subjects. The consistent superiority of RMETNet across both datasets and evaluation protocols further confirms its robustness and potential for practical BCI applications.

### Ablation Study

To assess the contributions of the two cross-domain components, namely the multi-scale Riemannian feature module and the MMD loss, ablation experiments were conducted on both datasets. Table 7 reports four settings: removing both components, removing only the multi-scale Riemannian feature module, removing only the MMD loss, and using the complete model. The ablation results indicate that the multi-scale Riemannian feature module and the MMD-based domain adaptation strategy provide complementary gains in RMETNet. Relative to the setting without either component, the Riemannian module alone increases average accuracy by 0.04% and 0.23% on BCICIV2a and BCICIV2b, respectively, whereas the MMD loss alone increases it by 2.41% and 1.12%. When both components are used together, the gains reach 4.28% on BCICIV2a and 1.89% on BCICIV2b. According to the paired Wilcoxon signed-rank tests, all three ablated settings are significantly worse than the full model on BCICIV2a (*p* < 0.01). On BCICIV2b, the settings without both components and without the MMD loss are significantly worse than the full model (*p* < 0.01), whereas the setting without the Riemannian module is not significantly different from the full model. These findings suggest that TSLANet and the spatio-temporal convolution module provide robust temporal and local spatial representations, while the multi-scale Riemannian branch captures global covariance structure across subjects. At the same time, the MMD loss encourages the network to learn more domain-invariant features by reducing cross-subject distribution differences. Together, these components improve the overall performance of RMETNet on both public datasets and support its potential for practical MI-BCI applications.

**Table 7:** Ablation study results on the BCICIV2a and BCICIV2b datasets.

Dataset	Methods		S1	S2	S3	S4	S5	S6	S7	S8	S9	Avg.	Kappa
	Riemannian	MMD loss											
BCICIV2a	×	×	74.28	51.23	81.34	58.30	56.32	55.42	76.56	77.41	73.09	67.11 ± 10.89^**^	0.55
	×	√	75.22	49.16	82.41	61.02	66.26	59.56	78.24	80.74	73.04	69.52±10.59^**^	0.58
	√	×	73.68	49.01	80.91	61.63	57.33	54.26	75.56	80.42	71.52	67.15±11.16^**^	0.55
	√	√	76.01	49.79	84.58	64.93	67.78	61.18	80.00	80.73	77.54	71.39±10.65	0.59
BCICIV2b	×	×	77.54	70.14	66.83	89.15	84.55	81.20	85.72	79.57	76.68	79.04±6.82^**^	0.51
	×	√	79.06	72.78	63.39	89.64	84.46	85.31	85.57	83.33	77.87	80.16±7.57	0.54
	√	×	77.20	72.22	65.35	90.11	84.41	81.68	86.00	79.98	76.46	79.27±7.08^**^	0.53
	√	√	81.88	73.21	66.70	91.63	85.74	83.36	86.90	81.71	77.20	80.93±7.12	0.54

The symbols ^*^ and ^**^ indicate statistically significant differences compared to the full model at the *p* < 0.05 and *p* < 0.01 levels, respectively, based on paired Wilcoxon signed-rank tests.

Although the standalone improvement brought by the multi-scale Riemannian feature module is relatively limited in the ablation study, its contribution should be understood together with the MMD-based domain adaptation strategy rather than in isolation. Specifically, the Riemannian module alone yields only marginal gains over the setting without both components, whereas the full model consistently achieves the best performance when the Riemannian feature learning and MMD loss are jointly applied. More importantly, on BCICIV2a, the benefit of the Riemannian module becomes evident when it is combined with MMD: adding the Riemannian branch to the MMD-based model increases the average accuracy from 69.52% to 71.39%, and the paired Wilcoxon signed-rank test confirms that this improvement is statistically significant (*p* < 0.01). This observation suggests that the main role of the Riemannian branch is not merely to provide an independent accuracy boost, but to supply geometry-aware covariance representations that are more amenable to cross-subject alignment under MMD regularization. In other words, the Riemannian module and the MMD loss appear to act synergistically: the former enhances the structural representation of EEG covariance patterns, while the latter promotes distribution matching across subjects in the learned feature space. This complementary interaction is particularly important in cross-subject MI decoding, where reducing inter-subject variability is as important as improving feature discriminability. Therefore, although the additional operations of covariance computation, logarithmic mapping, and tangent-space projection increase model complexity, the experimental results suggest that their value lies mainly in strengthening the effectiveness of domain adaptation rather than serving as a purely standalone enhancement. Future work will further investigate this synergy by analyzing computational cost, feature distributions, and transfer behavior under different subject-transfer settings.

### Visualization

**Fig 5:**
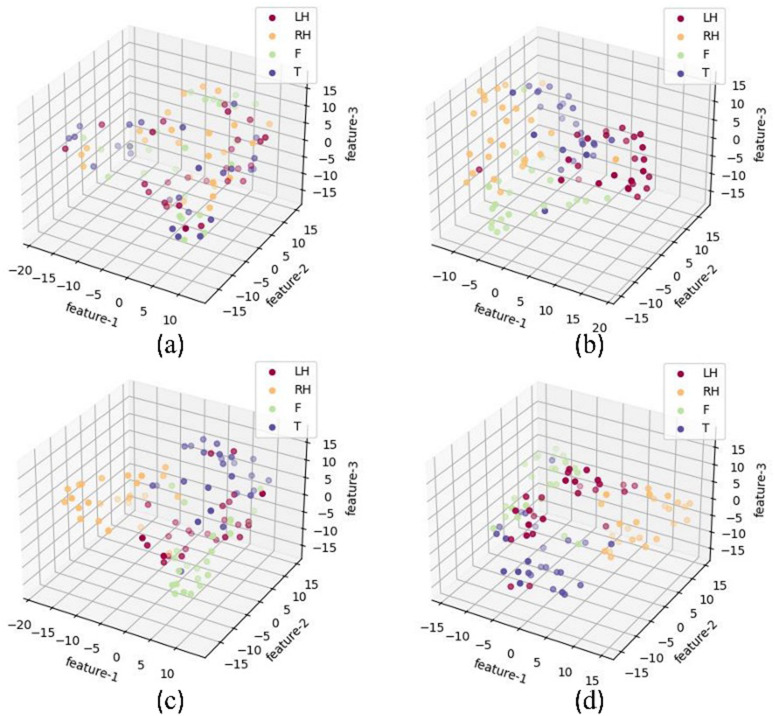
UMAP visualization of the test features for subject S1 on BCICIV2a under different settings. **(a)** Original test features. **(b)** Features without the Riemannian branch. **(c)** Features without MMD-loss-based training. **(d)** Features produced by the complete model.

UMAP [40] was used to visualize the deep feature distributions and to examine how the two cross-domain components affect class separation. Compared with t-SNE, UMAP preserves more global structure and therefore provides a clearer view of the learned feature organization. After training, the feature distribution of subject S1 on the BCICIV2a dataset is shown in Fig 5. Without the MMD loss and Riemannian feature fusion, the distances between classes are smaller. When the Riemannian branch is removed, the features show larger intra-class dispersion and weaker inter-class separation. By contrast, the complete model produces more compact intra-class clusters and clearer inter-class boundaries, which supports the effectiveness of RMETNet in learning discriminative subject representations.

### Complexity analysis

To evaluate the computational cost and deployment feasibility of RMETNet, we analyzed the model size, FLOPs, and single-trial inference latency. On BCICIV2a, RMETNet contains 0.198 M trainable parameters and requires 421.681 M FLOPs per trial; on BCICIV2b, it contains 0.159 M parameters and requires 57.752 M FLOPs. Under CPU-based single-trial inference, the average forward latency is 3.74 ± 0.04 ms on BCICIV2a and 0.52 ± 0.02 ms on BCICIV2b. In addition, covariance estimation and tangent-space alignment are performed during offline preprocessing rather than within the online forward pass, introducing only about 0.15 ms per trial for 22-channel data and 0.03 ms per trial for 3-channel data. These results indicate that RMETNet has a lightweight forward architecture and is suitable for near-real-time MI-BCI deployment. Nevertheless, because the cross-subject evaluation in this study relies on precomputed alignment statistics, the current experimental setting is more appropriately interpreted as session-level adaptation rather than fully streaming online inference.

## Conclusion

This study proposes RMETNet, a neural network for cross-subject motor imagery EEG decoding that combines TSLANet, spatio-temporal convolution, multi-scale Riemannian feature learning, and MMD-based domain adaptation. After Z-score normalization, the raw EEG signals are processed by TSLANet and the convolutional branch to extract temporal and spatio-temporal representations, while a parallel Riemannian branch learns cross-subject geometric features. In cross-subject experiments, RMETNet achieves average accuracies of 71.39% on BCICIV2a and 80.93% on BCICIV2b. In subject-dependent experiments, it reaches 80.71% and 86.76% on the two datasets, respectively, outperforming the compared deep learning baselines. Visualization and ablation analyses further confirm the effectiveness of the Riemannian feature mapping and the MMD-based cross-subject learning strategy. Future work will further optimize TSLANet for EEG analysis and investigate lighter model designs while preserving classification performance. Overall, RMETNet provides an effective and robust framework for MI-EEG-based BCI applications.
